# Genome-wide characterization of heavy metal-associated isoprenylated plant protein gene family from *Citrus sinensis* in response to huanglongbing

**DOI:** 10.3389/fpls.2024.1369883

**Published:** 2024-03-27

**Authors:** Guiyan Huang, Yanan Hu, Fuxuan Li, Xiru Zuo, Xinyou Wang, Fengyao Li, Ruimin Li

**Affiliations:** ^1^ College of Life Sciences, Gannan Normal University, Ganzhou, China; ^2^ China-USA Citrus Huanglongbing Joint Laboratory, National Navel Orange Engineering Research Center, College of Life Sciences, Gannan Normal University, Ganzhou, Jiangxi, China

**Keywords:** *Citrus sinensis*, heavy metal-associated isoprenylated plant proteins, phylogenetic analysis, susceptible gene, huanglongbing

## Abstract

**Introduction:**

Heavy metal-associated isoprenylated plant proteins (HIPPs) play vital roles in maintaining heavy metal balance and responding to both biotic and abiotic stresses in vascular plants. However, the role of HIPPs in the response to Huanglongbing (HLB), a harmful disease of citrus caused by the phloem-colonizing bacterium *Candidatus* Liberibacter asiaticus (CLas), has not been examined.

**Methods and results:**

In this study, a total of 26 *HIPP* genes were identified in *Citrus sinensis*, and they were grouped into 5 clades. The *CsHIPP* genes are distributed on 8 chromosomes and exhibited considerable synteny with *HIPPs* found *in Arabidopsis thaliana*. Additionally, we analyzed the gene structure, conserved motifs and domains of the CsHIPPs. Various cis-acting elements related to plant hormones and stress responses were identified in the promoters of *CsHIPPs*. Public transcriptome data and RT-qPCR analysis showed that the expression level of *CsHIPP03* was significantly reduced in samples infected by CLas and *Xanthomonas citri *ssp. *citri (Xcc)*. Furthermore, silencing the homologous gene of *CsHIPP03* in *Nicotiana benthamiana* increased the disease resistance of plants to bacteria.

**Discussion:**

Our results provide a basis for functional studies of *HIPP* gene family in *C. sinensis*, highlighting their functions in bacterial resistance, and improve our understanding to the susceptibility mechanism of HLB.

## Introduction

Heavy metal-associated isoprenylated plant proteins (HIPPs) are metal-binding metallochaperones consisting of one or two heavy metal-associated (HMA) domains and an isoprenylation motif (Barr et al., 2023). Moreover, glycine-rich or proline-rich regions are commonly found between the HMA domains and the isoprenylation motif (Suzuki et al., 2002; Barth et al., 2009). Genome-wide identification of HIPP members indicated various quantities of HIPPs in plants, as there were 45 members in *Arabidopsis thaliana*, 59 in *Oryza sativa*, 74 in *Populus trichocarpa*, 51 in *Setaria italica*, 5 in *Selaginella moellendorffii*, and 13 in *Fagopyrum tataricum* (de Abreu-Neto et al., 2013; Ye et al., 2022). Moreover, in Triticeae species, 114 HIPPs were identified in *Triticum aestivum*, 33 in *Triticum urartu*, 40 in *Aegilops tauschii*, 58 in *Triticum dicoccoides*, and 33 in *Hordeum vulgare* (Zhang et al., 2020c). The HIPPs were categorized into five clades through phylogenetic analysis, and segmental duplication played a significant role in the proliferation of the *HIPP* gene family (Khan et al., 2019; Zhang et al., 2020c).

The HMA domain contained a CXXC motif that was highly conserved, along with a βαββαβ-fold structure. This structure is responsible for binding Cu^2+^, Cd^2+^, or Zn^2+^ and is involved in the regulation of heavy metal homeostasis and detoxification (Dykema et al., 1999; Robinson and Winge, 2010). The C-terminus of the HIPPs usually includes a CaaX motif, which is crucial for its biological functions, including protein–membrane and protein–protein interactions (de Abreu-Neto et al., 2013). Research on HIPPs focuses on their connection to both abiotic and biotic stresses (Guo et al., 2021). *AtCdI19* in *A. thaliana* showed induction in response to Cd^2+^, Hg^2+^, Fe^2+^, and Cu^2+^ stress treatments, and its overexpression resulted in increased Cd tolerance in transgenic *Arabidopsis* (Suzuki et al., 2002). The expression of *A. thaliana HIPP20*, *HIPP22*, *HIPP26*, and *HIPP27* in yeast resulted in the enhancement of Cd resistance in the Cd-sensitive yeast strain *ycf1*. Nevertheless, the *A. thaliana hipp20/21/22* triple mutant exhibited heightened sensitivity to Cd (Tehseen et al., 2010). The heterologous expression of *VvHIPP21* from *Vitis vinifera* in *A. thaliana* resulted in decreased resistance to cold and drought in plants (Zheng et al., 2023). Furthermore, *A. thaliana HIPP5*, *HIPP6*, *HIPP7*, and *HIPP34* were implicated in plant endoplasmic reticulum-associated degradation (Guo et al., 2021). Various *HIPP* genes were targeted by pathogen effectors and served as susceptibility genes (Cowan et al., 2018; Radakovic et al., 2018). For example, the *potato mop-top virus* (PMTV) movement protein TGB1 interacted with NbHIPP26 to activate drought stress response genes and facilitate virus long-distance movement (Cowan et al., 2018). Knocking out of *AtHIPP27* in *A. thaliana* plants resulted in decreased susceptibility to beet cyst nematode (*Heterodera schachtii*) infection (Radakovic et al., 2018). Additionally, overexpression of *TaHIPP1* increased plant susceptibility to *Puccinia striiformis* f. sp. *Tritici*, while knocking down *TaHIPP1* expression enhanced wheat resistance (Zhang et al., 2015).

Susceptibility genes (*S*) are crucial for the pathogenicity and virulence of pathogens. It is believed that the products of *S* genes may be utilized by pathogens for host recognition, infiltration, acquisition of nutrients, proliferation, and transmission, or to suppress the host immune response (Langner et al., 2018; Zaidi et al., 2018). There were many *S* genes in different plants, like *elF4E* in *A. thaliana* (Pyott et al., 2016), *SlMlo1* in tomato (Nekrasov et al., 2017), *DIPM-1* in apple (Malnoy et al., 2016), *TaEDR1* in wheat (Zhang et al., 2017), *OsSWEET14* in rice (Li et al., 2012), and *CsLOB1* in citrus (Peng et al., 2017). Inactivating the *S* genes can disrupt the baseline susceptibility of hosts and result in disease resistance (Xu et al., 2019). A deleterious mutation in the *elF4E* gene locus of *A. thaliana* results in plants that are completely resistant to *Turnip mosaic virus* (Pyott et al., 2016). Rice resistance to bacterial blight was enhanced when the expression of *OsSWEET14* was suppressed (Li et al., 2012). By utilizing CRISPR/Cas9 technology, grapefruit and sweet orange were able to develop resistance to canker disease by editing the promoter and coding region of the citrus susceptibility gene *CsLOB1* (Peng et al., 2017).

Citrus huanglongbing (HLB) is a highly damaging disease that has led to a substantial decrease in citrus production on a global scale (Bové, 2006). In field, the Asian citrus psyllid (*Diaphorina citri*) transmit *C*Las to citrus while feeding on sap. Subsequently, *C*Las established itself in the phloem sieve element, ultimately causing disease symptoms (da Graca et al., 2016). As the citrus industry rapidly develops and the scale of citrus trade continues to expand, *C*Las and Asian citrus psyllid have spread globally (Wang, 2019). There are no effective strategies for curing HLB at the moment, and all citrus cultivars could potentially be affected by the HLB-associated bacterium *C*Las (Hu et al., 2021). It is difficult to uncover the pathogenic mechanism of *C*Las because it cannot be cultured *in vitro* (Pagliaccia et al., 2017). The *C*Las has a genome size of approximately 1.23 Mb and does not have type III and type IV secretion systems. However, it does have a complete type I secretion system and the general secretory pathway (GSP) (Duan et al., 2009). The GSP is crucial for transporting bacterial proteins, while the Sec-dependent effectors (SDEs) play a significant role in the infection of plants by pathogens (Liu et al., 2021). Researches have indicated that the immune responses of citrus can be influenced by several SDEs of *C*Las (Clark et al., 2018; Zhang et al., 2020b, 2023; Shi et al., 2023a, Shi et al., 2023b).

Previously, we had identified CsHIPP03 (namely, HIPP7) as a potential interactor of *C*Las core effector SDE34 in yeast two-hybrid (Y2H) screening (Hu et al., 2023). However, a thorough analysis of the *HIPP* gene family in citrus has not been documented. In this study, we identified 26 *HIPP* genes and systematically investigated the putative functions of *HIPP* genes in *C. sinensis*. Their detailed phylogenetic relationships, gene structures, subcellular localization, and expression profiles under *C*Las infection were investigated. Additionally, we performed a functional analysis of *CsHIPP03* in *Nicotiana benthamiana*. Our research has established a foundation for further examination of the role of *HIPP* genes in *C*Las-citrus interaction.

## Materials and methods

### Plant materials

The *A. thaliana* and *N. benthamiana* plants were cultivated in an artificial climate culture chamber under specific conditions (16/8 h day/night cycle, 23°C ± 2°C temperature, and 50%–60% relative humidity). The leaves and branches selected for gene cloning and expression analysis were obtained from healthy and *C*Las-infected *C. sinensis* cv. Newhall navel trees in Ganzhou, China. These trees were 3 years old and had Citrange rootstock.

### Identification of the HIPPs in *C. sinensis*

The protein sequences of *C. sinensis* (version 3.0) were obtained from the Citrus Pan-genome to Breeding Database (CPBD) (http://citrus.hzau.edu.cn/) (Liu et al., 2022). The protein sequences of *C. sinensis* with the HMA domain (PF00403.28) were searched using HMMER web server (Finn et al., 2011). Afterward, the candidate sequences were sent to the NCBI CDD database to search for conserved domains, and any sequences that included the HMA domain were retained (Marchler-Bauer et al., 2017). Subsequently, the reserved sequences that were predicted containing the isoprenylation motif CaaX using PrePS were recognized as HIPP proteins (Maurer-Stroh and Eisenhaber, 2005).

### Phylogenetic analysis

The protein sequences of HIPPs in *A. thaliana* were generated from a previous published study (de Abreu-Neto et al., 2013). The protein sequence of HIPPs in *A. thaliana* and *C. sinensis* was aligned using MAFFT (Katoh et al., 2019). Optimized partitioning scheme and evolutionary models were determined by PartitionFinder2 (Lanfear et al., 2017). Maximum likelihood phylogenetic analysis was inferred with IQ-TREE (Nguyen et al., 2015).

### Chromosomal distribution, synteny analysis, and exon–intron structural analysis

Chromosomal distribution of the *CsHIPP* genes was visualized using TBtools (Chen et al., 2020) with the GFF3 file downloaded from CPBD (Liu et al., 2022). The collinearity relationship of *HIPP* genes from *A. thaliana* and *C. sinensis* was conducted using MCScanX and BLASTP methods integrated in TBtools (Wang et al., 2012; Chen et al., 2020). Moreover, the exon–intron structure of *HIPP* genes were determined using the “Visualize Gene Structure” plug-in of TBtools (Chen et al., 2020).

### Conserved motifs and domain analysis

The conserved motif of HIPP proteins was assessed using MEME suite online (Bailey et al., 2015). Then, conserved domains of HIPP proteins were determined using “Batch CD-Search” tools in the NCBI CDD database (Marchler-Bauer et al., 2017).

### RNA-seq analysis

Released RNA-seq data related with HLB and citrus canker of *C. sinensis* in SRA database were downloaded and reanalyzed locally as mentioned previously with few modifications (Pertea et al., 2016). In brief, selected RNA-seq data were downloaded using the SRA Toolkit from the SRA database. Raw RNA-seq data were trimmed with Trimmomatic 0.36.0 (Bolger et al., 2014) and then transcript quantification was performed using the HISAT2-StringTie-ballgown RNA-seq pipeline (Fu et al., 2019). The reference genome of *C. sinensis* (version 3.0) for RNA-seq analysis was download from CPBD (Liu et al., 2022). The expression data of *HIPPs* were extracted and normalized using the *Z*-score method for heat map construction with the Pheatmap package (Kolde and Kolde, 2015). The BioSamples accessions of RNA-seq data used in this study are listed in **Supplementary Table 1**.

### RT-qPCR analysis

Total RNA of plant samples was extracted using the *EasyPure* ^®^ Plant RNA Kit (TransGen Biotech) according to the manufacturer’s protocol. First-strand cDNA was synthesized with *EasyScript* ^®^ All-in-One First-Strand cDNA Synthesis SuperMix for qPCR (One-Step gDNA Removal) (TransGen Biotech) and diluted 20 times for RT-qPCR using specific primers (**Supplementary Table 2**). Reactions were performed in a volume of 20 μL with 1× TransStart^®^ Tip Green qPCR SuperMix, using forward and reverse primers at concentrations of 0.2 μM. The PCR cycling began with an initial activation step at 98°C for 10 min, then proceeded with 40 cycles of 95°C for 15 s and 60°C for 40 s. All cDNA samples were run in triplicate. The *C. sinensis glyceraldehyde-3-phosphate dehydrogenase* (*GAPDH*) gene (NCBI Reference Sequence: XM_006468885.2) and *N. benthamiana actin* gene (NCBI Reference Sequence: AY179605.1) were used as endogenous controls. Relative expression levels were calculated using the 2^−ΔΔCT^ method (Livak and Schmittgen, 2001).

### Cis-acting elements analysis

The 2,000-bp upstream genomic sequences of *CsHIPP*s were obtained from the reference genome of *C. sinensis* (version 3.0) (Liu et al., 2022). The *cis*-acting elements in the promoter regions of *CsHIPPs* were *in silico* analysis with the PlantCARE database (Lescot et al., 2002).

### Vector construction

The plasmids were digested using the corresponding restriction enzymes. The gene fragments with homologous arms to the plasmids were amplified and subjected to homologous recombination with the linearized plasmids using the *pEASY* ^®^-T&B Simple Cloning Kit (TransGen Biotech, China). Subsequently, the recombinant plasmid was transformed into *Escherichia coli* (TOP10) chemically competent cells. Positive clones were identified by PCR and then confirmed by sequencing (Tsingke). The plasmids, restriction sites, and primers used in this study are listed in **Supplementary Table 2**, with details as follows. For the promoter activity assay, the promoter sequences of *CsHIPP03* were cloned and inserted into the plant binary vector pCAMBIA1380-GUS digested by *Sbf* I and *Xba* I to drive expression of β-glucuronidase (GUS). For subcellular localization, the coding sequences of *CsHIPP03*, *CsHIPP10*, *CsHIPP13*, *CsHIPP19*, *CsHIPP20*, *CsHIPP22*, *CsHIPP23*, and *CsHIPP26*, which do not contain stop codons, were cloned and inserted into the plant binary vector pCAMBIA2300-GFP digested by *Sac* I and *Xba* I. For Y2H assays, the coding sequences of *NbHIPP3.1* (*Niben101Scf07236g00011*), *NbHIPP3.2* (*Niben101Scf06423g01011*), and *NbHIPP3.3* (*Niben101Scf26202g00005*) from *N. benthamiana* were amplified and inserted into the prey vector pGADT7 digested with *EcoR* I and *BamH* I. For virus-induced gene silencing (VIGS) assays, 300-bp fragments of *NbHIPP3.1*, *NbHIPP3.2*, and *NbHIPP3.3* were cloned and inserted into vector pTRV2 digested with *EcoR* I and *BamH* I.

### Genetic transformation of *A. thaliana* and citrange hairy roots

P_HIPP03_-GUS and P_HIPPM_-GUS were transformed into *A. thaliana* through floral dipping method (Clough and Bent, 1998). In addition, these vectors were transformed into *Agrobacterium rhizogenes* (K599), and the Carrizo citrange hairy roots were transformed by the *A. rhizogenes*-mediated method as described previously (Ma et al., 2022). The transformed hairy roots were identified by GFP fluorescence observation.

### GUS staining

The transgenic *A. thaliana* and citrange hairy roots were used for GUS staining and observed using an optical microscope. In brief, plant tissue samples were submerged in GUS staining buffer (Coolaber, China) and stained for a period of 8 h. Subsequently, samples were decolorized by repeated immersion in 70% alcohol three times prior to photography.

### Subcellular localization

*A. tumefaciens* strain GV3101 (pMP90) cells containing binary vectors were cultured overnight in Luria Bertani liquid medium with 20 μg mL^−1^ rifampicin and 50 μg mL^−1^ kanamycin and resuspended in MES solution (10 mM MgCl_2_, 10 mM MES, pH 5.6, and 200 mM acetosyringone). The cultures were diluted to OD_600_ of 0.4. The diluted *A. tumefaciens* suspension was used to infiltrate the leaves of *N. benthamiana*, which were then incubated in darkness at 22°C for 2 days. Subsequently, the fluorescence signals were observed using a Leica Sp8 confocal microscope (Huang et al., 2019).

### Yeast two-hybrid assays

BD-SDE34 and AD-NbHIPPs were co-transformed into yeast Y2Hgold strain, and the resultant diploid yeasts were cultured on synthetic dropout medium (SD)/-Leu/-Trp (DDO) and SD/-Leu/-Trp/-His (TDO) agar plates supplemented with 40 μg/mL X-a-gal to monitor the interaction. Images were captured 3 days post-plating.

### TRV-based VIGS in *N. benthamiana*

*A. tumefaciens* strain GV3101 (pMP90) cultures carrying the TRV-RNA1 plasmid and TRV-RNA2 cassettes were grown overnight, collected by centrifugation at 3,500 rpm for 5 min, and then resuspended in MES solution to a final OD_600_ of 0.5. The TRV-RNA2 and TRV-RNA1 solutions were mixed in equal volumes and then infiltrated into the abaxial side of two *N. benthamiana* leaves on seedlings at the four-leaf stage. Three weeks post-infection, RT-qPCR was carried out to detect the VIGS efficiency. *Pseudomonas syringae* pv. *tomato* DC3000 mutant Δ*hopQ1-1* were inoculated at 1.0 × 10^4^ colony-forming units (CFU) mL^−1^ for virulence assays (Wei et al., 2007). In order to assess bacterial growth, the 1-cm^2^ leaf discs from *N. benthamiana* were ground in 1 ml of sterile water, and then serial dilutions were spotted onto KB medium. CFU were counted 2 days after incubation at 28°C (Ramos et al., 2021).

### Statistical analysis

The statistical significance of the results was assessed by conducting a two-sided, unpaired Student’s *t*-test using SPSS 25.0. The significance levels were indicated as follows: * for *p* < 0.05, ** for *p* < 0.01, and *** for *p* < 0.001.

## Results

### Identification and characterization of the HIPP members in *C. sinensis*

After hmmsearch using the HMM profile of HMA (PF00403.28) and confirmation using the NCBI CDD database, 26 genes encoding HIPP proteins containing the HMA domain and isoprenylation motif were identified in the genome of *C. sinensis*. The protein lengths of CsHIPPs ranged from 101 to 554 amino acids, with the molecular weight ranging from 11.43 to 59.91 kDa. The isoelectric point of CsHIPPs ranged from 4.96 to 10.16 and the grand average of hydropathy value varied from −1.215 to −0.118 (**Supplementary Table 3**).

### Phylogenetic analysis of the HIPP members

**Figure 1:**
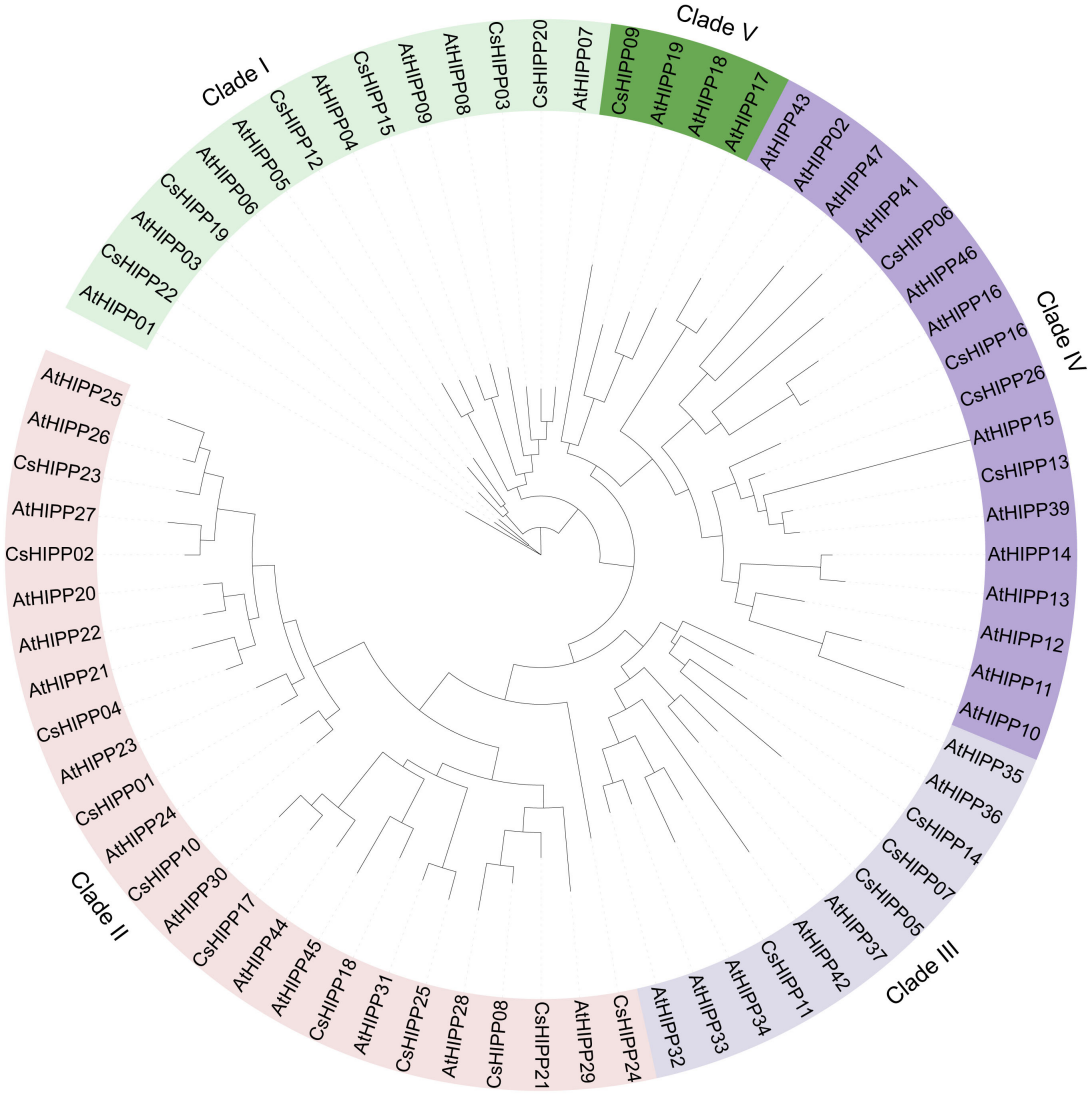
The phylogenetic analysis of the HIPPs from *Citrus sinensis* and *Arabidopsis thialina*. The phylogenetic tree contains 71 HIPPs, which were classified into five clades, namely, 26 HIPPs from *C. sinensis* and 45 HIPPs from *A. thialina*. Each clade’s label is positioned adjacent to the respective protein identifiers.

To reveal the phylogeny of HIPP proteins, the 26 CsHIPPs and 45 AtHIPPs were phylogenetically analyzed and a Maximum likelihood tree was built using IQ-TREE (**Figure 1**). HIPP members were divided into five clades based on the topology structure of the phylogenetic tree. The minimum clade, clade 4, consisted of four HIPP proteins, namely, CsHIPP09, AtHIPP17, AtHIPP18, and AtHIPP19, while the maximum clade, clade 2, contained 25 HIPP proteins, namely, 11 CsHIPPs and 14 AtHIPPs. In detail, clade 1 contained 6 CsHIPPs and 8 AtHIPPs, clade 3 contained 4 CsHIPPs and 7 AtHIPPs, and clade 5 contained 4 CsHIPPs and 13 AtHIPPs (**Figure 1**).

### Chromosomal distribution and synteny analysis of the HIPP members

**Figure 2:**
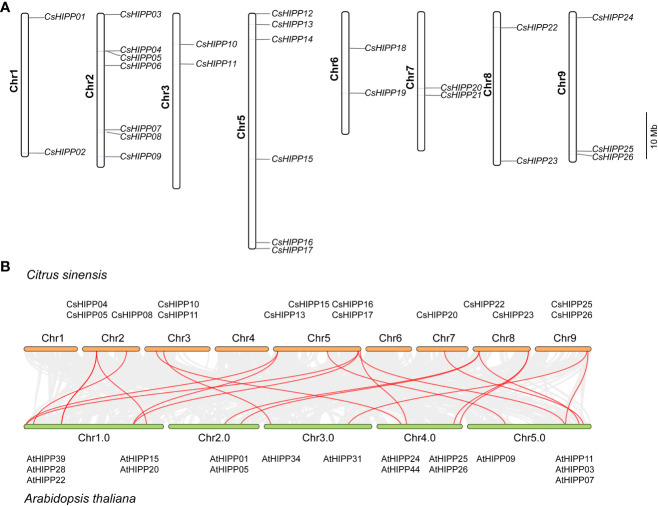
Chromosomal distribution of the *CsHIPP* genes and the collinearity relationships of *CsHIPPs* and *AtHIPPs*. **(A)** Chromosomal distribution of the *HIPPs* from *C. sinensis.* The identities of the chromosomes are marked on the left side of each chromosome, while the *HIPP* gene names are shown to the right of each chromosome. The scale on the right is in megabases (Mb). **(B)** The collinearity relationships between the *CsHIPP* genes and *AtHIPP* genes. Red lines indicate the existence of collinear relationship between the two, with a total of 21 collinear events.

According to the gene location annotation information, the chromosomal distribution of *CsHIPPs* was performed. As shown in **Figure 2**, the HIPP members distributed separately on eight out of nine chromosomes of *C. sinensis* except chromosome 4. Chromosome 2 contained seven *CsHIPPs*, chromosome 5 contained six *CsHIPPs*, chromosome 9 contained three *CsHIPPs*, and there are two *CsHIPPs* distributed on each of the other remaining chromosomes (**Figure 2A**). Moreover, the collinearity relationships between the *CsHIPPs* and *AtHIPPs* were investigated. Twenty-one collinearity events occurred. Particularly, 14 *CsHIPPs* had collinearity relationships with 19 *AtHIPPs* (**Figure 2B**).

### Gene structure analysis of *CsHIPPs*

**Figure 3:**
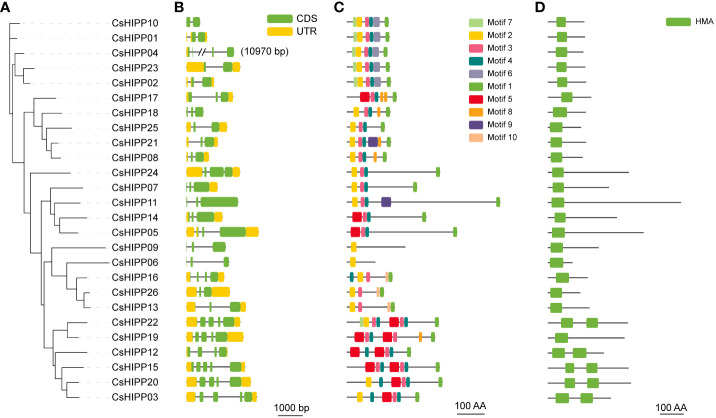
The gene structure, motif, and conserved domain analysis of CsHIPPs. **(A)** Phylogenetic tree of CsHIPPs. **(B)** The exons/introns structure of *CsHIPP* genes (green indicates CDSs, yellow indicates upstream/downstream sequences, and black line indicates introns). **(C)** Conservation motif analysis of CsHIPPs. **(D)** HMA conservative domain of CsHIPPs.

The gene structures of *CsHIPPs* were explored to understand the connections between gene structure and gene function (**Figure 3**). The number of exons in *CsHIPPs* ranged from 2 to 5. Thirteen *CsHIPPs* contained three exons, which is the primary gene structure of *CsHIPPs*, and five *CsHIPPs* had five exons, four *CsHIPPs* had four exons, and four *CsHIPPs* had two exons (**Figure 3B**). The phylogenetic relationships of CsHIPPs (**Figure 3A**) were consistent with their gene structures (**Figure 3B**).

### Conserved motifs and domains analysis of the HIPP members in *C. sinensis*

A total of 10 conserved motifs and one conserved domain were identified from CsHIPPs (**Figure 3C**). Similar to the clusters in **Figure 1**, CsHIPPs were divided into five clades (**Figure 3A**, **Supplementary Table 3**). All clade I members contained motifs 1, 3, 4 and 5. Clade II and III members shared motifs 1, 3, and 4. In addition, clades IV and V shared motif 2. Most of the CsHIPPs had more than four motifs except CsHIPP06 and CsHIPP09, which contained only one motif 4. Moreover, motif 9 presents only in CsHIPP11 and CsHIPP21 (**Figure 3C**). All CsHIPPs shared a conserved domain “HMA”. However, Clade I members contained two HMA domains except CsHIPP19, and the other clade members contained only one HMA domain (**Figure 3D**).

### Response of *CsHIPPs* to HLB and citrus canker by transcriptome analysis

**Figure 4:**
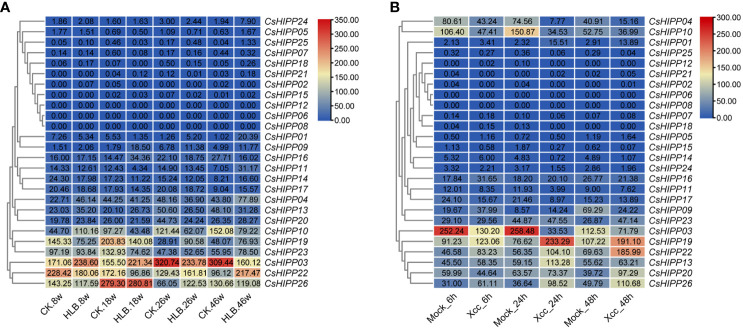
Expression profiles of the *CsHIPP* genes in different periods after infection by *Candidatus* Liberobacter asiaticus (*C*Las) **(A)** and *Xanthomonas citri* ssp. *citri* (*Xcc*) **(B)**. “CK” and “Mock” represent control samples, while “HLB” signifies *C*Las-infected samples and “*Xcc*” refers to samples infected with *Xcc*. The durations post-CLas infection were 8 weeks, 18 weeks, 26 weeks, and 46 weeks, denoted as 8w, 18w, 26w, and 46w, respectively. The durations post-Xcc infection were 6 h, 24 h, and 48 h, denoted as 6h, 24h, and 48h, respectively. The numbers within the rectangle represent the transcripts per million value for each gene in each sample.

To understand the dynamic expression profiles of *CsHIPPs* in response to HLB and citrus canker, the two major diseases in citrus industry, the expression quantity of *CsHIPPs* was generated by reanalyzing related RNA-seq data in the SRA database. In the progress of *C*Las infection, the expression tendency of *CsHIPPs* varied irregularly. However, three genes (e.g., *CsHIPP03*) downregulated when *C*Las-infected samples in contrast to *C*Las-free samples at the 26- and 46-week time points. In addition, nine *CsHIPPs* upregulated in *C*Las-infected samples at the 46-week time point (**Figure 4A**). In response to citrus canker, eight *CsHIPPs* (e.g., *CsHIPP01*) upregulated after citrus bacterial canker infected for 24 and 48 h. Thirteen *CsHIPPs* (e.g., *CsHIPP03*) downregulated when citrus bacterial canker infected for 24 h while nine *CsHIPPs* (e.g., *CsHIPP10*) downregulated for 48 h (**Figure 4B**). Interestingly, two *CsHIPPs*, *CsHIPP03* and *CsHIPP10*, showed downregulation in response to the infection of *C*Las and citrus bacterial canker (**Figure 4**).

### Gene expression of *CsHIPPs* in response to *C*Las infection

**Figure 5:**
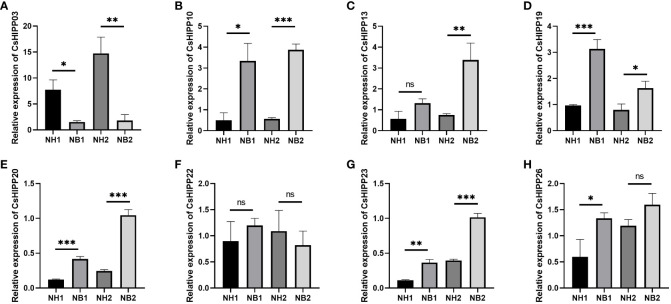
RT-qPCR analysis of eight *CsHIPP* genes in healthy and huanglongbing-positive *C. sinensis*. Relative expression of **(A)** *CsHIPP03*, **(B)** *CsHIPP10*, **(C)** *CsHIPP13*, **(D)** *CsHIPP19*, **(E)** *CsHIPP20*, **(F)** *CsHIPP22*, **(G)** *CsHIPP23*, and **(H)** *CsHIPP26*. Asterisks indicate that the corresponding genes were distinctly up- or downregulated in different samples by *t*-test (*p < 0.05, **p < 0.01, ***p < 0.001). The citrus *GAPDH* gene was used as internal reference, three biological replicates were adopted, and the relative expression of *CsHIPP* genes was calculated by the 2^−ΔΔCT^ method. NB: Newhell *C*Las infection, NH: Newhall *C*Las free, 1: young leaves, 2: mature leaves. ns, not significant.

Many studies have shown that the HIPPs play an important role in the phytopathogenic–bacterial interaction system. To further validate the expression changes of *CsHIPPs* under the induction of HLB, we selected eight genes, namely, *CsHIPP03*, *CsHIPP10*, *CsHIPP13*, *CsHIPP19*, *CsHIPP20*, *CsHIPP22*, *CsHIPP23*, and *CsHIPP26*, for expression pattern analysis. The expression levels were detected by RT-qPCR in the midrib tissues of *C*Las-free and -infected *C. sinensis* cv. Newhall. Results showed that after *C*Las infection, the expression of *CsHIPP03* was significantly downregulated in young and mature tissues (**Figure 5A**). Furthermore, with the exception of CsHIPP22 showing no significant difference in expression level, the other six genes exhibited upregulation during HLB infection (**Figure 5**).

### *Cis*-acting elements distribution in the promoter region of *CsHIPPs*

Promoters are pivotal in regulating gene expression, whereas *cis*-acting elements in the promoter region served as binding sites for associated transcription factors (Hernandez-Garcia and Finer, 2014). To understand the dynamic expression patterns of *CsHIPPs* when citrus was infected with *C*Las and *Xanthomonas citri* ssp. *citri* (*Xcc*), the *cis*-acting elements distributed on the upstream genomic region of *CsHIPPs* were searched and those related with phytohormone, abiotic, and biotic stress responsiveness were reserved. As shown in **Supplementary Figure 1**, abscisic acid responsiveness, auxin responsiveness, defense and stress responsiveness, DRE, gibberellin responsiveness, low-temperature responsiveness, MeJA responsiveness, and salicylic acid responsiveness dispersedly distributed on the promoter region of *CsHIPPs*. Twenty-two *CsHIPPs* contained the abscisic acid responsiveness elements, and *CsHIPP10*, which encompassed six, had the most abscisic acid responsiveness elements. In addition, 22 *CsHIPPs* had the MeJA responsiveness elements. Moreover, 16 *CsHIPPs* contained the gibberellin responsiveness elements. Fourteen *CsHIPPs* contained the salicylic acid responsiveness elements. Twelve *CsHIPPs* contained the auxin responsiveness elements. Eight *CsHIPPs* contained the low-temperature responsiveness elements. Seven *CsHIPPs* contained the defense and stress responsiveness elements. It is worth noting that only *CsHIPP03* and *CsHIPP23* contained the DRE elements (**Supplementary Figure 1**). In short, *CsHIPPs* could response to various phytohormone, abiotic, and biotic stress while each *CsHIPP* had its unique *cis*-acting element array mode.

### Promoter activity of *CsHIPP03*

**Figure 6:**
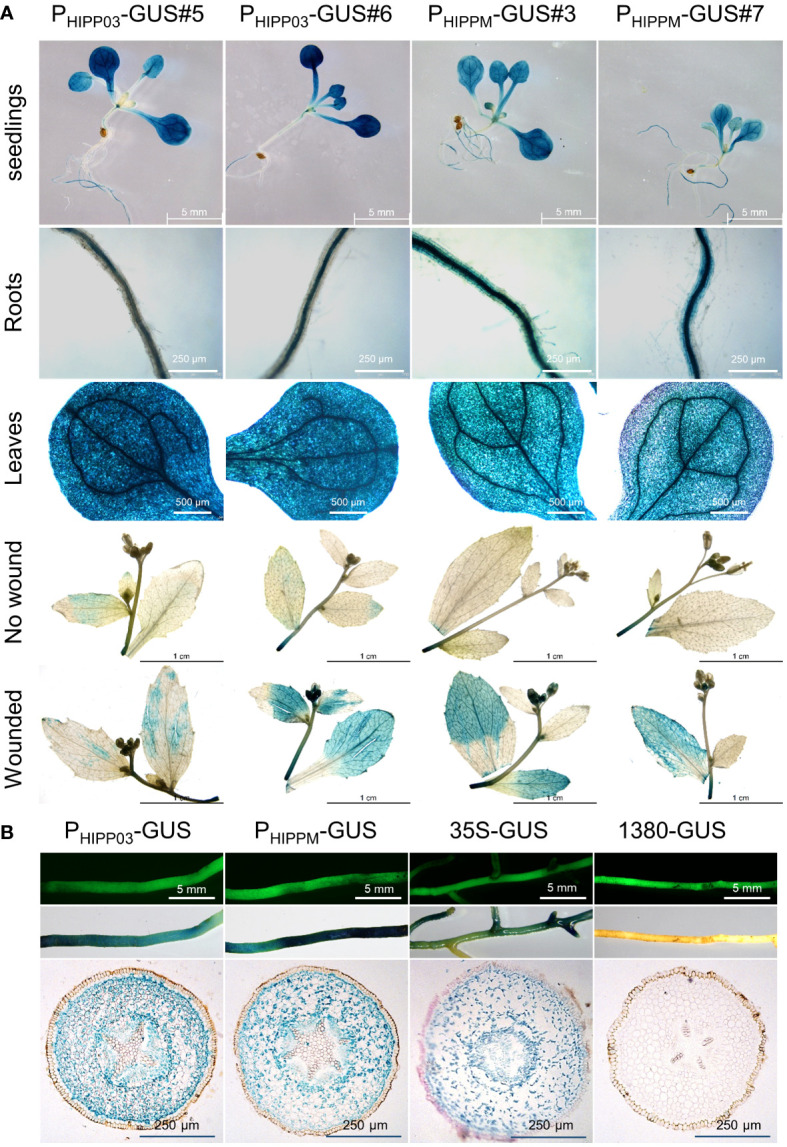
*CsHIPP03* is induced by wound and expressed in different tissues. **(A)** GUS staining of seedlings and mature plants with or without wound of P_HIPP03_-GUS and P_HIPPM_-GUS transgenic *A. thaliana*. **(B)** GUS staining and GFP fluorescence observation of transgenic citrange hairy roots. GFP fluorescence indicates that the root has been successfully transformed. P_HIPP03_ represents the promoter that matches the reference genome sequence and P_HIPPM_-GUS represents a mutat with gaps and mismatches.

It has been reported that *HIPP26* from *A. thaliana* and *N. benthamiana* is highly expressed in plant vascular tissues (Barth et al., 2009; Cowan et al., 2018). Since the phloem is the specific colonization site for *C*Las, we were interested in that whether CsHIPP03, a target of *C*Las (Hu et al., 2023), is expressed in vascular tissues of citrus. To assess the promoter activity of *CsHIPP03*, a 2,287-bp sequence upstream of the gene was cloned into the vector pCAMBIA1380-GUS to construct P_HIPP03_-GUS. Additionally, a mutated sequence including 149-bp gaps and 11 mismatches was identified during sequencing, which was named P_HIPPM_-GUS. Both P_HIPP03_-GUS and P_HIPPM_-GUS constructs were transformed into *A. thaliana* and citrange hairy roots to investigate their activity. GUS staining of different developmental stages and tissues showed that both promoters were active in the leaves and roots of *A. thaliana* seedlings, with higher expression in leaf veins. Furthermore, they were significantly induced by wound treatment in mature *A. thaliana* plants (**Figure 6A**). In transgenic citrange, GUS staining revealed that blue staining was observed in the cortex, columnar sheath, and phloem of the hairy roots, but not in the xylem. This was different from the 35s promoter (**Figure 6B**).

### Subcellular localization analysis of CsHIPPs

**Figure 7:**
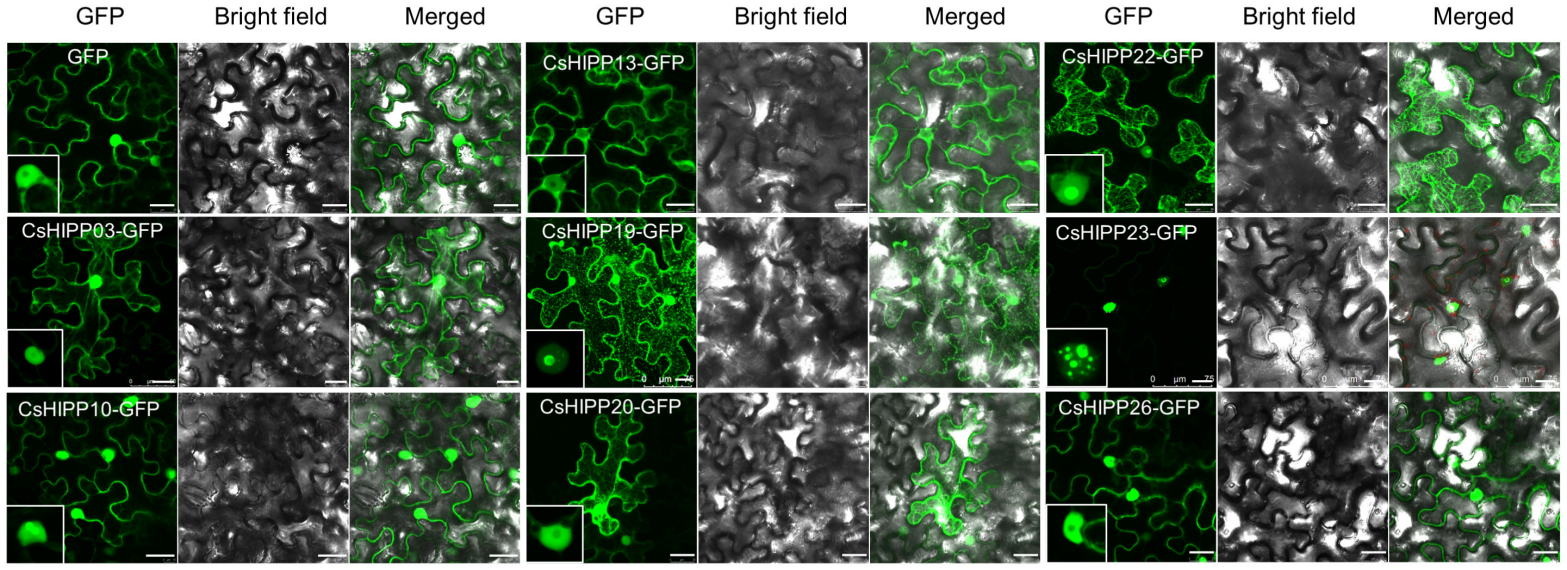
Subcellular localization of CsHIPPs in *Nicotiana benthamiana.* Subcellular localization of CsHIPP03-GFP, CsHIPP10-GFP, CsHIPP13-GFP, CsHIPP19-GFP, CsHIPP20-GFP, CsHIPP22-GFP, CsHIPP23-GFP, and CsHIPP26-GFP. GFP was used as a control. The insert images display the nucleus. Bar = 25 μm.

To understand the subcellular localization of CsHIPPs, we selected eight CsHIPPs (namely, CsHIPP03, CsHIPP10, CsHIPP13, CsHIPP19, CsHIPP20, CsHIPP22, CsHIPP23, and CsHIPP26) for further analysis. The results showed that all CsHIPPs, with the exception of CsHIPP23, were found in the plasma membrane (PM), although they exhibited varying levels of fluorescence intensity (**Figure 7**). The fluorescence intensity of CsHIPP10, CsHIPP19, CsHIPP22, and CsHIPP23 was stronger in the nucleolus than others. CsHIPP23 showed many spot-like fluorescence signals in the nucleus. In addition, CsHIPP19 and CsHIPP22 are located in the nucleus and microtubule, and there are dotted signals at the junction of microtubules. Interestingly, we found that overexpression of CsHIPP26 would stimulate plant necrosis in *N. benthamiana* (**Supplementary Figure 2**).

### Silencing of *NbHIPP3* enhances the *N. benthamiana* resistance to *P. syringae*

**Figure 8:**
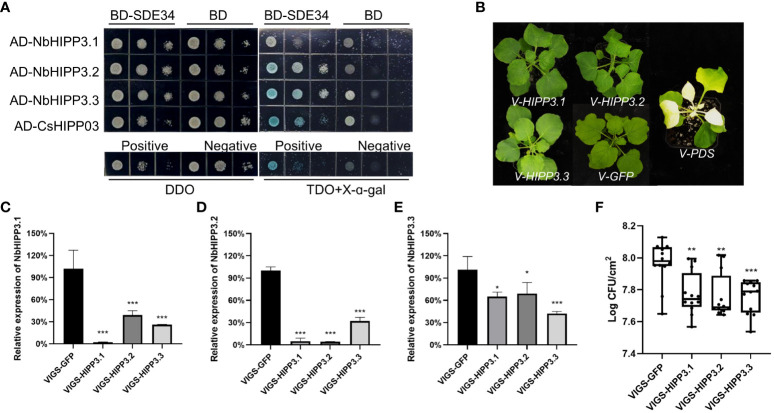
Silencing of *NbHIPP3* genes inhibits the colonization of *Pto DC3000ΔhopQ1-1* in *Nicotiana benthamiana* leaves. **(A)** SDE34 interacted with NbHIPP3.1, NbHIPP3.2, and NbHIPP3.3 by yeast two-hybrid. DDO indicates synthetic dropout medium (SD)/-Leu/-Trp and TDO indicates SD/-Leu/-Trp/-His. Positive control, pGBKT7-53 + pGADT7-T; Negative control, pGBKT7-Lam + pGADT7-T. **(B)** The growth phenotype of *N. benthamiana* after virus induced silencing of *NbHIPP3.1*, *NbHIPP3.2*, and *NbHIPP3.3*, with *GFP* and *PDS* as control. Relative expression of the **(C)** *NbHIPP3.1*, **(D)** *NbHIPP3.2*, and **(E)** *NbHIPP3.3* after gene silencing by VIGS. There were six biological replicates for each experimental group, with TRV-GFP as a control. Total RNAs were extracted from the upper leaves 20 dpi and were used for RT-qPCR. The *N. benthamiana* actin gene was used as endogenous controls. Asterisks indicate significant downregulation of genes by *t*-test (*p < 0.05, **p < 0.01, ***p < 0.001). **(F)** The colonization of *Pto DC3000ΔhopQ1-1* was lower in *NbHIPP3.1*, *NbHIPP3.2*, and *NbHIPP3.3* gene-silenced *N. benthamiana* leaves. This experiment were repeated three times with consistent results. * denotes *p* < 0.05, ** denotes *p* < 0.01, and *** denotes *p* < 0.001.

To verify whether CsHIPP03, the target of SDE34 (Hu et al., 2023), is a negative immune regulator, a tobacco rattle virus-based system was used to knock down the expression of *NbHIPP3* in *N. benthamiana*. Through sequence alignment, we identified three homologous genes of Cs*HIPP03* in *N. benthamiana*, named *NbHIPP3.1*, *NbHIPP3.2*, and *NbHIPP3.3* (**Supplementary Figure 3**). Y2H assay indicated that SDE34 interacted with NbHIPP3.2 and NbHIPP3.3 (**Figure 8A**). Subsequently, we designed a 300-bp silencing sequence for each homologous gene, and these fragments were cloned into TRV2 in an antisense orientation respectively, and we conducted VIGS experiments to determine the role of NbHIPP3s in the bacterial resistance of *N. benthamiana*. VIGS results showed that expression of the *NbHIPP3s* was reduced in inoculated plants compared with the control (TRV-GFP) (**Figure 8B**). Among them, the silencing efficiency of *NbHIPP3.1* and *NbHIPP3.2* is as high as 90% (**Figures 8C, D**), and *NbHIPP3.3* also has a silencing efficiency of approximately 60% (**Figure 8E**). Next, the VIGS plants were inoculated with the *P. syringae* pv. *tomato* DC3000 mutant Δ*hopQ1-1*, which can cause disease in *N. benthamiana* (Wei et al., 2007). The symptoms and the level of bacterial colonization were determined 2 days later. The results showed that compared with the control group (TRV-GFP), silencing *NbHIPP3s* significantly reduced the bacterial colonization (**Figure 8F**). In conclusion, silencing homologous genes of *CsHIPP03* in *N. benthamiana* enhanced the disease resistance.

## Discussion

HIPPs are key proteins involved in widespread biological processes such as transport of metallic ions, cold and drought stresses, and plant–pathogen interactions (de Abreu-Neto et al., 2013). Genome-wide identification and characterization analysis of the *HIPP* gene family have been implemented in *A. thaliana*, *O. sativa*, *P. trichocarpa*, *S. italica*, *S. moellendorffii*, *F. tataricum*, *T. aestivum*, *T. urartu*, *A. tauschii*, *T. dicoccoides*, and *H. vulgare* (de Abreu-Neto et al., 2013; Zhang et al., 2020c; Ye et al., 2022). In the present study, we performed a systematic analysis of the *HIPP* gene family in *C. sinensis*. A total of 26 HIPP members were identified in the genome of *C. sinensis*, and the number is less than HIPPs of other plants like 45 for *Arabidopsis*, 59 for rice, 74 for poplar, and 51 for millet (de Abreu-Neto et al., 2013). The difference could be on account of gene duplication events during polyploidization or whole genome duplication. The 26 HIPPs members in *C. sinensis* were divided into five clusters when compared to the phylogenetic analysis of HIPPs in *A. thaliana*, which is consistent with the cluster analysis in *O. sativa* (Khan et al., 2019). The protein conserved domains are responsible for biological functions of proteins in cells. Two types of proteins with the HMA domain have been discovered in plants: heavy metal-associated plant proteins (HPPs) and HIPPs (Barth et al., 2009; Tehseen et al., 2010). The difference is that HIPPs contain an isoprenylation motif on the C-terminus end. All identified *C. sinensis* HIPPs have one or two HMA domains and an isoprenylation motif. Interestingly, Clade I HIPPs of *C. sinensis* contain two HMA domains except for CsHIPP19 while the other HIPPs contain only one HMA domain. Similarly, the same cluster of HIPPs have a similar distribution of motifs.

Even though HIPPs have a significant impact on the metabolic regulation of metallic ions, we are paying close attention to their roles in citrus–bacterial interactions. The public transcriptome data in the NCBI SRA database related with HLB- or *Xcc*-infected samples were used for the dynamic expression analysis of *CsHIPPs* (Chin et al., 2021; Tang et al., 2021). *CsHIPP09* and *CsHIPP16* were significantly induced when *C. sinensis* was infected by *C*Las for 18 weeks. Alternatively, *CsHIPP01*, *CsHIPP04*, *CsHIPP11*, *CsHIPP21*, *CsHIPP24*, and *CsHIPP25* were significantly induced while *CsHIPP03*, *CsHIPP10*, and *CsHIPP16* were downregulated at 46 weeks. As for *Xcc*-infected samples, *CsHIPP21*, *CsHIPP19*, and *CsHIPP26* were significantly upregulated at 24 and 48 h. Conversely, *CsHIPP03*, *CsHIPP04*, *CsHIPP14*, *CsHIPP15*, *CsHIPP24*, and *CsHIPP25* were downregulated at 24 and 48 h. *CsHIPP03* were downregulated during the process of HLB and *Xcc* infection. In consideration of CsHIPP03 as a target of the effector from *C*Las, the function of CsHIPP03 in the interaction between citrus and *C*Las needs to be understood. VIGS assays were used to silence the *CsGAPCs* analogues in *N. benthamiana*, resulting in increased resistance to *Phytophthora capsici* infection. This finding supports the postulated role of *CsGAPCs* in citrus as a potential suppressor of immunity, suggesting a function that is conserved across disparate species (Shi et al., 2023b). Since the effector protein SDE34 of *C*Las can interact with CsHIPP03 (Hu et al., 2023), we speculate that CsHIPP03 plays an important role in the citrus–HLB interaction system. Furthermore, VIGS demonstrated that the knockdown of *NbHIPP3* increased the resistance of plants to bacteria, suggesting that *CsHIPP03* may be a susceptibility gene for HLB in citrus. However, further research is needed to confirm its biological function.

HIPP proteins were localized on the PM, including AtFP6 from *Arabidopsis* (Gao et al., 2009), NbHIPP26 from *N. benthamiana* (Cowan et al., 2018), OsHIPP29 from *O. sativa* (Zhang et al., 2020a), and HIPP1-V from *H. villosa* (Zhang et al., 2020c). The isoprenylation motif is an integral part of the HIPP protein structure, and any mutations in this motif may lead to changes in the protein’s localization. For instance, the AtIPT3 farnesylation directed its localization to the cytoplasm and nucleus, whereas the nonfarnesylated protein was found in the plastids (Galichet et al., 2008). Similarly, wild-type CdI19 was localized to the PM, whereas the farnesylation motif mutant was found in the entire cytoplasm (Suzuki et al., 2002). In our study, apart from CsHIPP23, all HIPP proteins from *C. sinensis* were localized on the PM. Notably, CsHIPP22 was also found to be localized on microtubules, like the co-localization result of TGB1 and NbHIPP26. However, NbHIPP26 is localized in the PM, nucleus, and motile vesicles (Cowan et al., 2018). In addition, during the cloning of CsHIPP22, a conserved sequence containing an intron was obtained, but the test results showed that the intron sequence did not affect its subcellular localization in *N. benthamiana.* Protein localization is related to function. The richness of the subcellular localization of citrus HIPP family indicates its functional diversity, but its specific function is still unknown. The HMA domain is known to be involved in heavy metal transport and maintenance of homeostasis, and the isoprenylation motif has a direct impact on the function of HIPPs. For instance, HIPP1-V, a positive regulator of powdery mildew resistance in *H. villosa*, was observed to be rapidly induced by *Blumeria graminis* f. sp. *tritics* (Bgt). Transiently or stably heterologous overexpression of HIPP1‐V in wheat was found to suppress the haustorium formation and enhance powdery mildew resistance, with isoprenylation being critical for PM localization, interaction with E3‐ligase CMPG1‐V, and function in powdery mildew resistance (Wang et al., 2023).

## Conclusion

In this study, we identified 26 *CsHIPPs* in *C. sinensis* genome and performed a systematic analysis of the CsHIPP gene family, including phylogenetic relationship, chromosome location, conserved motifs and domain, *cis*-acting elements, and expression pattern analysis. RT-qPCR analysis showed that the expression levels of six among the eight *CsHIPPs* were increased, and only the expression level of *CsHIPP03* gene was decreased. Further research has shown that silencing the expression of *NbHIPP3*, homologous genes of *CsHIPP03*, increased the resistance of *N. benthamiana* to *P. syringae*. The comprehensive analysis of the *CsHIPPs* at a genome-wide level will lay the groundwork for functional examinations of *CsHIPPs*. Furthermore, exploring the functions of *HIPPs* will aid in gaining insight into their biological roles.

## Data availability statement

The datasets presented in this study can be found in online repositories. The names of the repository/repositories and accession number(s) can be found in the article/**Supplementary Material**.

## Author contributions

GH: Funding acquisition, Investigation, Methodology, Software, Writing – original draft, Writing – review & editing. YH: Investigation, Methodology, Software, Writing – original draft. FuL: Funding acquisition, Methodology, Writing – original draft. XZ: Investigation, Software, Writing – original draft. XW: Investigation, Software, Writing – original draft. FeL: Investigation, Software, Writing – original draft. RL: Conceptualization, Funding acquisition, Project administration, Writing – original draft, Writing – review & editing.
