# Supplementary material for: Genome-wide characterization of heavy metal-associated isoprenylated plant protein gene family from Citrus sinensis in response to huanglongbing
Source: Front Plant Sci. 2024 Mar 27;15:1369883. doi: 10.3389/fpls.2024.1369883 (PMC11004388; doi:10.3389/fpls.2024.1369883)
Supplement: Supplementary file 1 [file DataSheet_1.docx]

Supplementary Materials

**Supplementary Table 1 |** BioSamples in SRA database used in this study.

| Accession | Samples | Links | Reference |
| --- | --- | --- | --- |
| SRX3605625 | CK.8w | https://www.ncbi.nlm.nih.gov/sra/SRX3605625 | <https://doi.org/10.1021/acs.jproteome.9b00616> |
| SRX3605626 |  | https://www.ncbi.nlm.nih.gov/sra/SRX3605626 |  |
| SRX3605627 |  | https://www.ncbi.nlm.nih.gov/sra/SRX3605627 |  |
| SRX3605628 |  | https://www.ncbi.nlm.nih.gov/sra/SRX3605628 |  |
| SRX3605621 | HLB.8W | https://www.ncbi.nlm.nih.gov/sra/SRX3605621 |  |
| SRX3605622 |  | https://www.ncbi.nlm.nih.gov/sra/SRX3605622 |  |
| SRX3605623 |  | https://www.ncbi.nlm.nih.gov/sra/SRX3605623 |  |
| SRX3605624 |  | https://www.ncbi.nlm.nih.gov/sra/SRX3605624 |  |
| SRX3605629 | CK.18w | https://www.ncbi.nlm.nih.gov/sra/SRX3605629 |  |
| SRX3605630 |  | https://www.ncbi.nlm.nih.gov/sra/SRX3605630 |  |
| SRX3605635 |  | https://www.ncbi.nlm.nih.gov/sra/SRX3605635 |  |
| SRX3605636 |  | https://www.ncbi.nlm.nih.gov/sra/SRX3605636 |  |
| SRX3605631 | HLB.18W | https://www.ncbi.nlm.nih.gov/sra/SRX3605631 |  |
| SRX3605632 |  | https://www.ncbi.nlm.nih.gov/sra/SRX3605632 |  |
| SRX3605637 |  | https://www.ncbi.nlm.nih.gov/sra/SRX3605637 |  |
| SRX3605638 |  | https://www.ncbi.nlm.nih.gov/sra/SRX3605638 |  |
| SRX3605639 | CK.26w | https://www.ncbi.nlm.nih.gov/sra/SRX3605639 |  |
| SRX3605640 |  | https://www.ncbi.nlm.nih.gov/sra/SRX3605640 |  |
| SRX3605633 |  | https://www.ncbi.nlm.nih.gov/sra/SRX3605633 |  |
| SRX3605634 |  | https://www.ncbi.nlm.nih.gov/sra/SRX3605634 |  |
| SRX3605647 | HLB.26W | https://www.ncbi.nlm.nih.gov/sra/SRX3605647 |  |
| SRX3605648 |  | https://www.ncbi.nlm.nih.gov/sra/SRX3605648 |  |
| SRX3605649 |  | https://www.ncbi.nlm.nih.gov/sra/SRX3605649 |  |
| SRX3605650 |  | https://www.ncbi.nlm.nih.gov/sra/SRX3605650 |  |
| SRX3605643 | CK.46w | https://www.ncbi.nlm.nih.gov/sra/SRX3605643 |  |
| SRX3605644 |  | https://www.ncbi.nlm.nih.gov/sra/SRX3605644 |  |
| SRX3605645 |  | https://www.ncbi.nlm.nih.gov/sra/SRX3605645 |  |
| SRX3605646 |  | https://www.ncbi.nlm.nih.gov/sra/SRX3605646 |  |
| SRX3605619 | HLB.46W | https://www.ncbi.nlm.nih.gov/sra/SRX3605619 |  |
| SRX3605620 |  | https://www.ncbi.nlm.nih.gov/sra/SRX3605620 |  |
| SRX3605641 |  | https://www.ncbi.nlm.nih.gov/sra/SRX3605641 |  |
| SRX3605642 |  | https://www.ncbi.nlm.nih.gov/sra/SRX3605642 |  |
| SRX9866661 | Mock_6h | https://www.ncbi.nlm.nih.gov/sra/SRX9866661 | https://doi.org/10.1371/journal.pgen.1009316 |
| SRX9866662 |  | https://www.ncbi.nlm.nih.gov/sra/SRX9866662 |  |
| SRX9892361 | Xcc_6h | https://www.ncbi.nlm.nih.gov/sra/SRX9892361 |  |
| SRX9892362 |  | https://www.ncbi.nlm.nih.gov/sra/SRX9892362 |  |
| SRX9893067 | Mock_24h | https://www.ncbi.nlm.nih.gov/sra/SRX9893067 |  |
| SRX9893068 |  | https://www.ncbi.nlm.nih.gov/sra/SRX9893068 |  |
| SRX9893139 | Xcc_24h | https://www.ncbi.nlm.nih.gov/sra/SRX9893139 |  |
| SRX9893140 |  | https://www.ncbi.nlm.nih.gov/sra/SRX9893140 |  |
| SRX9893072 | Mock_48h | https://www.ncbi.nlm.nih.gov/sra/SRX9893072 |  |
| SRX9893073 |  | https://www.ncbi.nlm.nih.gov/sra/SRX9893073 |  |
| SRX9893172 | Xcc_48h | https://www.ncbi.nlm.nih.gov/sra/SRX9893172 |  |
| SRX9893173 |  | https://www.ncbi.nlm.nih.gov/sra/SRX9893173 |  |

**Supplementary Table 2 |** Primers used in this study.

| Primer Name | Sequence (5'-3') | Usage |
| --- | --- | --- |
| Q-CsHIPP03-F1 | ATATGCATTGTGAAGCTTGTGCT | RT-qPCR |
| Q-CsHIPP03-R1 | CACACCTTTATCGGGTCTGC |  |
| Q-CsHIPP20-F | GTTGAGCTGCTGTCTCCGAT |  |
| Q-CsHIPP20-R | TTCTTCTCCTCAGGCTTCGG |  |
| Q-CsHIPP19-F | ATAACCACGTGTACGGCCAG |  |
| Q-CsHIPP19-R | TGAGGAGAGACGAGGTCCA |  |
| Q-CsHIPP23-F | GATGCGAGCGCAAGGTAAAG |  |
| Q-CsHIPP23-R | CGACGACCGTGACTTTGTTG |  |
| Q-CsHIPP10-F | GAAGGCACCATCGAGTACATATC |  |
| Q-CsHIPP10-R | AGCCATCACAATCCATCCTTAC |  |
| Q-CsHIPP13-F | CCATGGACATGAAGGAGAAGAA |  |
| Q-CsHIPP13-R | TTATATCTGTCGTCGGCCAATAC |  |
| Q-CsHIPP22-F | GGAGAGCAGGATGGAGTATTTC |  |
| Q-CsHIPP22-R | CCAACAGGGTACCCATATTCAT |  |
| Q-CsHIPP26-F | GGACATTGATCCGGTGAGTATAG |  |
| Q-CsHIPP26-R | CCTTCTTCTTCTCAGGCTCTTT |  |
| Q-GAPDH-F | CAGCACTCAAAGGCAAACTAAA |  |
| Q-GAPDH-R | GGACACCTGAACGACAAGAT |  |
| Q-NbHIPP3.1-F | GGAAGTAACGGCGGATTGTA |  |
| Q-NbHIPP3.1-R | GGCTTAGGCAAAGGTGAAATAAG |  |
| Q-NbHIPP3.2-F | ACAAGAGAACCGGGAAGCAA |  |
| Q-NbHIPP3.2-R | TCATCTTCTCCTTTGCTTTCTTC |  |
| Q-NbHIPP3.3-F | TAATAACGGTTGTCTTGAGCGT |  |
| Q-NbHIPP3.3-R | CAATGACACCTTTTACGACAAC |  |
| Q-NbAct-F | ACCATCAATGATCGGAATGG |  |
| Q-NbAct-R | GCTCATCCTATCAGCAATGC |  |
| VIGS-NbHIPP3.1-F | TGAGTAAGGTTACCGAATTCTATTTCACCTTTGCCTAAGCCA | Construction of TRV2 vector (EcoR I + BamH I) for VIGS |
| VIGS-NbHIPP3.1-R | GTGAGCTCGGTACCGGATCCTCCTCTTTCACTATTATAGCTTG |  |
| VIGS-NbHIPP3.2-F | TGAGTAAGGTTACCGAATTCATCCCTCAAAGGTTTCCAAGGA |  |
| VIGS-NbHIPP3.2-R | GTGAGCTCGGTACCGGATCCCTCACTAACTTTTCTGGATCAAC |  |
| VIGS-NbHIPP3.3-F | TGAGTAAGGTTACCGAATTCAGAAAAGTTGTTAGATCCTTGAAA |  |
| VIGS-NbHIPP3.3-R | GTGAGCTCGGTACCGGATCCATTACCGGAGGACGCAGAG |  |
| Pro-CsHIPP03-F | CATGCAAGCTTGCATGCCTGCAGGCTCACATGCTATGAGCTGATAG | Construction of pCambia1380-GUS vector (Sbf I + Xba I) for GUS staining and promoter activity assay |
| Pro-CsHIPP03-R | GTAACATCTCGAGGATCCTCTAGATATGTTTGTGATGATCCTAAAACAA |  |
| GFP-CsHIPP03-F | AGAACACGGGGGACGAGCTCATGGGTGAGGAGAACAAAGAG | Construction of pCambia2300GFP vector (Sac I + Xba I) for subcellular localization |
| GFP-CsHIPP03-R | ACCATGGTGTCGACTCTAGACATAACAAAGCAAGCATTTGGAT |  |
| GFP-CsHIPP20-F | AGAACACGGGGGACGAGCTCATGGGGGAGGAAGAAAAGAAG |  |
| GFP-CsHIPP20-R | ACCATGGTGTCGACTCTAGACATTACAGAACAGGCATTAGGGT |  |
| GFP-CsHIPP19-F | AGAACACGGGGGACGAGCTCATGGGCGAGCAAAATGAGGGA |  |
| GFP-CsHIPP19-R | ACCATGGTGTCGACTCTAGACATAACAGAACACGCGTTGG |  |
| GFP-CsHIPP23-F | AGAACACGGGGGACGAGCTCATGGGTGTTGTGGATCATTTTTC |  |
| GFP-CsHIPP23-R | ACCATGGTGTCGACTCTAGACATGATGGCGCAGGCTTGC |  |
| GFP-CsHIPP10-F | AGAACACGGGGGACGAGCTCATGGGAGTCGAAGGCACCATC |  |
| GFP-CsHIPP10-R | ACCATGGTGTCGACTCTAGACATGATTGAGCACGCATTAGGATT |  |
| GFP-CsHIPP13-F | AGAACACGGGGGACGAGCTCATGAAGAAGGCTGTATTAAAGTTGG |  |
| GFP-CsHIPP13-R | ACCATGGTGTCGACTCTAGAACAAATAACACAAGCGTTTGGATTC |  |
| GFP-CsHIPP22-F | AGAACACGGGGGACGAGCTCATGGGCAAGAAGAAGAAGAACAAC |  |
| GFP-CsHIPP22-R | ACCATGGTGTCGACTCTAGACATAACAACACAGGCATTCGGG |  |
| GFP-CsHIPP26-F | AGAACACGGGGGACGAGCTCATGAAGAAAGCAGTGTTGAAACTTG |  |
| GFP-CsHIPP26-R | ACCATGGTGTCGACTCTAGAGCAAATAACACAAGCATTAGGATC |  |
| NbHIPP3-1-F | ATGGCCATGGAGGCCAGTGAATTCATGGGTGAGGAGAACAAAGG | Construction of pGADT7 vector (EcoR I + BamH I) for yeast two hybrid |
| NbHIPP3-1-R | CTGCAGCTCGAGCTCGATGGATCCTTACATGACAGAGCAAGCATGA |  |
| NbHIPP3-2-F | ATGGCCATGGAGGCCAGTGAATTCATGGGTGAGGAAAAGGAGAAC |  |
| NbHIPP3-2-R | CTGCAGCTCGAGCTCGATGGATCCTTACATGACAGAGCAAGCATGA |  |
| NbHIPP3-3-F | ATGGCCATGGAGGCCAGTGAATTCATGGGTGAGGAGAAGAAAGTG |  |
| NbHIPP3-3-R | CTGCAGCTCGAGCTCGATGGATCCTTACATGAGAGAGCACGCATGA |  |

**Supplementary Table 3 |** Analysis of physicochemical properties of CsHIPP proteins.

| Gene Name | Gene Locus | CDS (bp) | Amino acids (aa) | MW (kDa) | Conserved Domain | pI | GRAVY | Subcellular location | Clade |
| --- | --- | --- | --- | --- | --- | --- | --- | --- | --- |
| *CsHIPP01* | *Cs_ont_1g000950* | 462 | 153 | 16.76 | HMA/CaaX | 9.89 | -0.466 | Chloroplast | II |
| *CsHIPP02* | *Cs_ont_1g028570* | 477 | 158 | 18.14 | HMA/CaaX | 9.32 | -0.634 | Nucleus | II |
| *CsHIPP03* | *Cs_ont_2g000480* | 786 | 261 | 29.55 | HMA/CaaX | 7.19 | -1.038 | Mitochondrion/Nucleus | I |
| *CsHIPP04* | *Cs_ont_2g012550* | 441 | 146 | 16.34 | HMA/CaaX | 10.16 | -0.534 | Chloroplast | II |
| *CsHIPP05* | *Cs_ont_2g012560* | 1197 | 398 | 43.30 | HMA/CaaX | 8.43 | -0.772 | Nucleus | III |
| *CsHIPP06* | *Cs_ont_2g016210* | 306 | 101 | 11.43 | HMA/CaaX | 8.50 | -0.202 | Chloroplast/Cytoplasm/Nucleus | V |
| *CsHIPP07* | *Cs_ont_2g025960* | 762 | 253 | 28.21 | HMA/CaaX | 8.45 | -0.760 | Nucleus | III |
| *CsHIPP08* | *Cs_ont_2g026570* | 432 | 143 | 16.24 | HMA/CaaX | 8.32 | -0.756 | Nucleus | II |
| *CsHIPP09* | *Cs_ont_2g032860* | 633 | 210 | 23.68 | HMA/CaaX | 8.59 | -0.758 | Nucleus | IV |
| *CsHIPP10* | *Cs_ont_3g010420* | 453 | 150 | 16.58 | HMA/CaaX | 10.14 | -0.417 | Chloroplast | II |
| *CsHIPP11* | *Cs_ont_3g016580* | 1665 | 554 | 59.91 | HMA/CaaX | 9.51 | -1.215 | Nucleus | III |
| *CsHIPP12* | *Cs_ont_5g000080* | 696 | 231 | 26.43 | HMA/CaaX | 9.87 | -0.455 | Chloroplast | I |
| *CsHIPP13* | *Cs_ont_5g003680* | 519 | 172 | 19.50 | HMA/CaaX | 7.50 | -1.104 | Nucleus | V |
| *CsHIPP14* | *Cs_ont_5g008590* | 861 | 286 | 31.07 | HMA/CaaX | 8.12 | -0.943 | Nucleus | III |
| *CsHIPP15* | *Cs_ont_5g030100* | 1008 | 335 | 37.90 | HMA/CaaX | 4.96 | -0.858 | Nucleus | I |
| *CsHIPP16* | *Cs_ont_5g048550* | 495 | 164 | 18.80 | HMA/CaaX | 9.72 | -0.893 | Nucleus | V |
| *CsHIPP17* | *Cs_ont_5g050630* | 540 | 179 | 20.58 | HMA/CaaX | 9.32 | -0.427 | Chloroplast | II |
| *CsHIPP18* | *Cs_ont_6g012110* | 471 | 156 | 18.25 | HMA/CaaX | 5.01 | -0.424 | Nucleus | II |
| *CsHIPP19* | *Cs_ont_6g021360* | 957 | 318 | 34.72 | HMA/CaaX | 9.33 | -0.953 | Nucleus | I |
| *CsHIPP20* | *Cs_ont_7g012650* | 1038 | 345 | 38.21 | HMA/CaaX | 5.66 | -1.085 | Nucleus | I |
| *CsHIPP21* | *Cs_ont_7g013590* | 477 | 158 | 18.81 | HMA/CaaX | 8.08 | -0.962 | Nucleus | II |
| *CsHIPP22* | *Cs_ont_8g005760* | 999 | 332 | 35.99 | HMA/CaaX | 9.50 | -1.157 | Nucleus | I |
| *CsHIPP23* | *Cs_ont_8g027910* | 465 | 154 | 17.33 | HMA/CaaX | 9.70 | -0.519 | Chloroplast/Nucleus | II |
| *CsHIPP24* | *Cs_ont_9g002150* | 1011 | 336 | 37.39 | HMA/CaaX | 6.32 | -1.162 | Nucleus | II |
| *CsHIPP25* | *Cs_ont_9g025690* | 411 | 136 | 15.17 | HMA/CaaX | 7.03 | -0.118 | Cytoplasmic | II |
| *CsHIPP26* | *Cs_ont_9g026520* | 402 | 133 | 14.93 | HMA/CaaX | 9.09 | -0.826 | Nucleus | V |


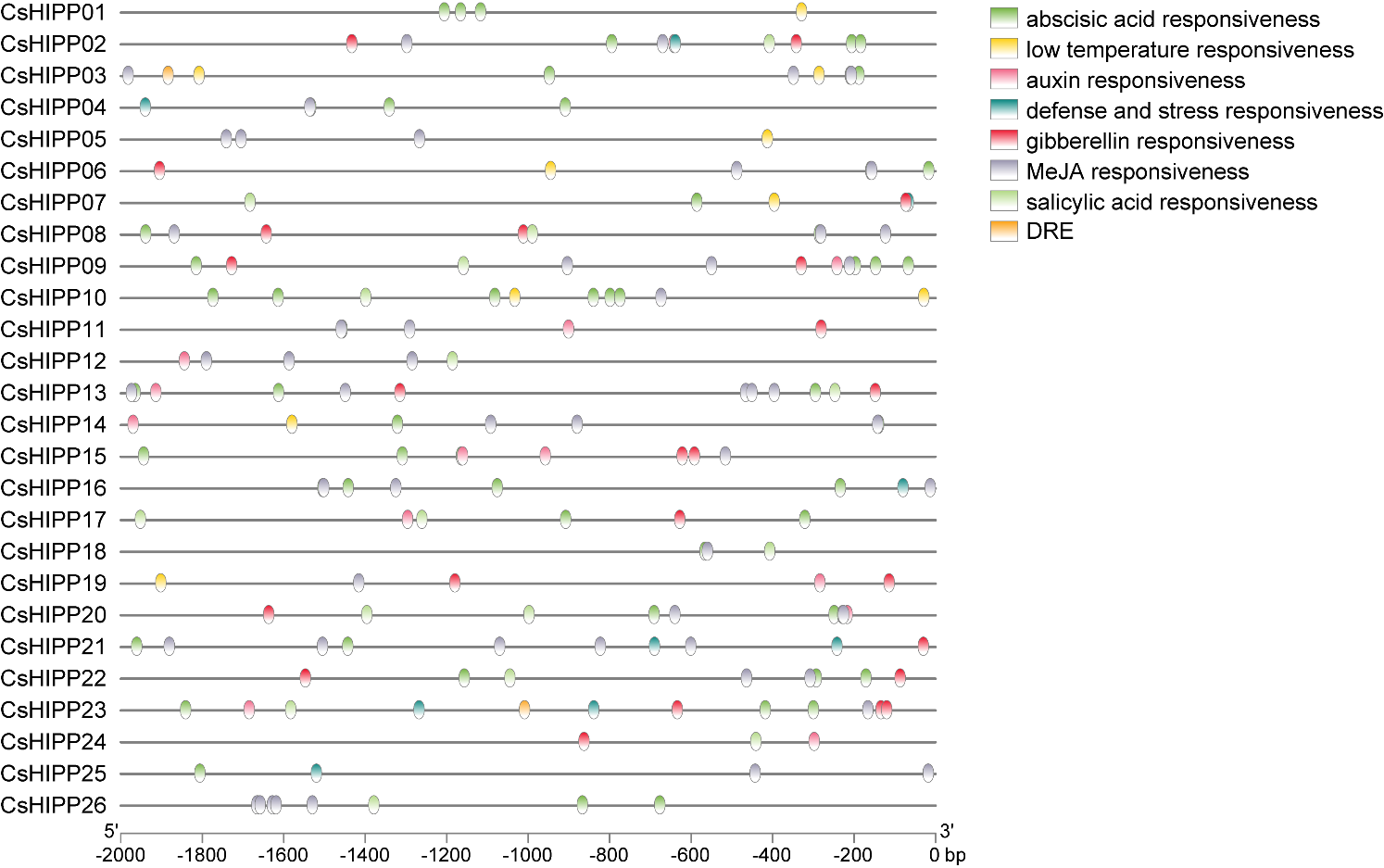


**Supplementary Figure 1 |** The *cis*-acting elements distribution in the promoter region of the 26 *CsHIPP* genes. The *cis*-acting elements in promoters of the 26 *CsHIPP* genes were predicted using PlantCARE online (http://bioinformatics.psb.ugent.be/webtools/plantcare/html/). Each *cis*-acting element was demarcated with a distinct color label.


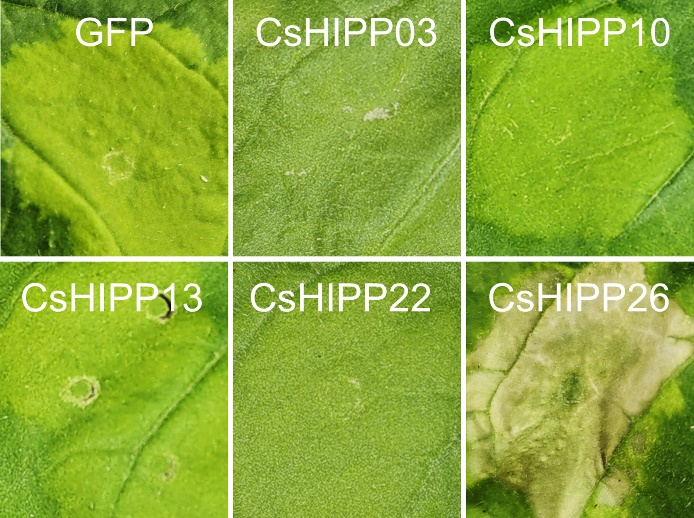


**Supplementary Figure 2 |** Overexpression of *CsHIPP26* resulted in cell necrosis in *Nicotiana benthamiana*. Transient expression of *CsHIPP03*, *CsHIPP10*, *CsHIPP13*, *CsHIPP22*, and *CsHIPP26*, using GFP as a control, reveals that the overexpression of *CsHIPP26* induces cell necrosis in *N. benthamiana*.

**
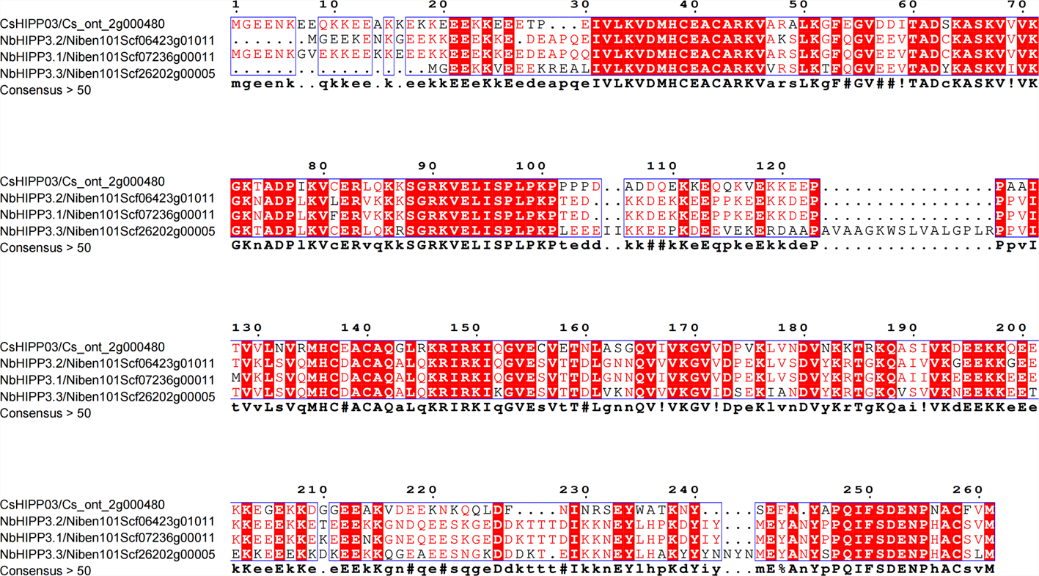
**

**Supplementary Figure 3 |** Sequence alignment of CsHIPP03 homologous proteins in *Nicotiana benthamiana*. Three homologous proteins of CsHIPP03, namely, NbHIPP3.1 (Niben101Scf07236g00011), NbHIPP3.2 (Niben101Scf06423g01011), and NbHIPP3.3 (Niben101Scf26202g00005), are identified in the genome of *Nicotiana benthamiana* version 1.01 (https://solgenomics.net/organism/Nicotiana_benthamiana/genome). The sequence alignment was performed using MAFFT online (https://www.ebi.ac.uk/jdispatcher/msa/mafft) and displayed using ESPript 3.0 online (https://espript.ibcp.fr/ESPript/ESPript/).
